# Modelling the power threshold and optimum thermal deformation of indirectly liquid-nitro­gen cryo-cooled Si monochromators

**DOI:** 10.1107/S1600577524002133

**Published:** 2024-04-09

**Authors:** Hossein Khosroabadi, Lucia Alianelli, Pablo Sanchez-Navarro, Andrew Peach, Kawal Sawhney

**Affiliations:** a Diamond Light Source, Harwell Science and Innovation Campus, Didcot, Oxfordshire OX11 0DE, United Kingdom; b MAX IV Laboratory, Fotongatan 2, 224 84 Lund, Sweden; Australian Synchrotron, Australia

**Keywords:** cryo-cooled Si monochromator, deformation modelling, threshold power, sweet spot, cooling design

## Abstract

Maximizing the performance of crystal monochromators is a key aspect in the design of beamline optics for diffraction-limited synchrotron sources. Temperature and deformation of cryo-cooled crystals, illuminated by high-power beams of X-rays, can be estimated with a purely analytical model. The analysis is based on the thermal properties of cryo-cooled silicon crystals and the cooling geometry. Deformation amplitudes can be obtained, quickly and reliably. In this article the concept of threshold power conditions is introduced and defined analytically. The contribution of parameters such as liquid-nitro­gen cooling efficiency, thermal contact conductance and interface contact area of the crystal with the cooling base is evaluated. The optimal crystal illumination and the base temperature are inferred, which help minimize the optics deformation. The model has been examined using finite-element analysis studies performed for several beamlines of the Diamond-II upgrade.

## Abstract 2

A robust theoretical analysis of thermal management of cryo-cooled Si monochromators in hard X-ray beamlines is developed. This universal theory has been validated by extensive finite-element analysis studies, offering guidances to assess the heatload deformation quickly.

## 1. Introduction

Cryo-cooled Si crystals (Marot *et al.*, 1992 ▸; Bilderback *et al.*, 2000 ▸) are commonly used as hard X-ray monochromators in synchrotron beamlines (Lee *et al.*, 2000 ▸, 2001 ▸; Mochizuki *et al.*, 2001 ▸; Zhang *et al.*, 2003 ▸; Chumakov *et al.*, 2004 ▸). The thermal deformation induced by high heat load is successfully minimized using the appropriate cooling design (Zhang *et al.*, 2013 ▸; Huang & Bilderback, 2012 ▸; Huang *et al.*, 2014 ▸). Increased beam brightness and collimation in new low-emittance synchrotron machines is driving progress to further control deformation and stability of double-crystal monochromators (DCMs). The optics cooling is constantly evaluated with the aim of improving the thermal response to photon beams with higher power (Brumund *et al.*, 2021 ▸; Chumakov *et al.*, 2014 ▸; Liu *et al.*, 2016 ▸; Petrov *et al.*, 2022 ▸; Zhang *et al.*, 2023 ▸; Liang *et al.*, 2018 ▸; Rebuffi *et al.*, 2020 ▸; Qin *et al.*, 2022 ▸; Wu *et al.*, 2021 ▸).

Finite-element analysis (FEA) studies are regularly carried out to assess the functionality of white-beam slits and DCMs at Diamond Light Source (DLS). Power (*P*) and power spatial density (*P*_d_) absorbed by the optics will increase considerably on the upgraded machine Diamond-II (D-II) (Chapon *et al.*, 2019 ▸). Installation of cryo-cooled or hybrid permanent-magnet undulators (CPMUs, HPMUs) with higher magnetic field will contribute to such an increase. Power management is key to conserving the photon source brightness on the lower-emittance machines.

The design of suitable DCM cooling is a complex and multi-parameter problem. Power levels are not constant on a given beamline, due to changing of settings, such as the insertion device gap, the angular fan of the incident beam, the optical layout, the presence of filters and the crystal Bragg angle. Exhaustive FEA is normally performed to study a few of these power scenarios and to finalize the cooling geometry. However, Si crystal temperature or thermal distortion do not follow a simple linear trend with *P* or *P*_d_, making interpretation and extrapolation of the FEA results complicated. In an earlier study an analytical model was developed (Khosroabadi *et al.*, 2022 ▸), which describes the universal behaviour of cryo-cooled Si deformation and the transition between concave, flat and convex regimes. The results from the model agree both with available experimental data (Lee *et al.*, 2001 ▸; Khosroabadi *et al.*, 2022 ▸) and FEA data (Zhang *et al.*, 2013 ▸, 2023 ▸; Huang & Bilderback, 2012 ▸; Huang *et al.*, 2014 ▸; Liu *et al.*, 2016 ▸).

This article, which is an extension of the study already published by Khosroabadi *et al.* (2022 ▸), offers a practical and simplified treatment of parameters affecting crystal deformation. A threshold line is calculated for the space of possible parameters *P* and *P*_d_. Crystal deformation is acceptable below the threshold line, whilst it reaches a critical regime and is difficult to control above the line. In addition, the optimum temperature for the Si crystal base and the copper cooling block are provided; these ensure a minimized surface deformation for any values of *P* and *P*_d_ below the threshold.

## 2. Theoretical model

The theoretical model (Khosroabadi *et al.*, 2022 ▸) is summarized and extended to obtain a threshold power and the so-called ‘sweet spot’ condition. The crystal temperature distribution is calculated using the crystal base temperature *T*_b_ as a boundary condition. *T*_b_ can be either measured by a thermocouple attached to the crystal or derived analytically as shown below. For a circular footprint, we use the radial symmetry of the problem, and derive the temperature *T*(*r*) inside the crystal by solving the heat transfer conduction equation (Yener & Kakaç, 2008 ▸),

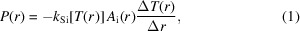

where *k*_Si_ is the thermal conductivity of Si, *P*(*r*) is the absorbed power, and *A*_i_ = 2π*r* ^2^ is the interface area at distance *r*. At low power, *k*_Si_ is assumed to be constant, and *T*(*r*) is

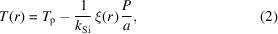

where

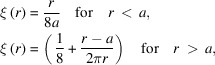

where *T*_p_ = *T*(0) is the crystal peak temperature and *a* is the radius of the beam footprint. For high power, *k*_Si_ has an inverse quadratic temperature dependence, and so a complicated exponential function of temperature is derived (Khosroabadi *et al.*, 2022 ▸). For medium-energy synchrotron machines, with electron beam energy *E*_e_ ≃ 3 GeV, the following linear equations are good approximations,

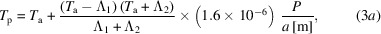




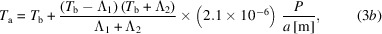




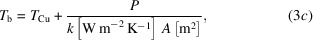

where Λ_1_ = 34 K and Λ_2_ = 158 K. *T*_Cu_, *A* and *k* are, respectively, the average temperature of the Cu block, the contact area and the thermal conductance at the copper–silicon contact surface. Units used hereafter are W, W mm^−2^ and K, for *P*, *P*_d_ and *T*, respectively. The solution of equations 3(*a*)–3(*c*) shows that *T*_p_ (which dictates the crystal deformation) has a complex dependence on power, beam footprint size, the cooling coefficients and finally the cooling geometry which determines the *T*_Cu_ and *T*_b_ values.

If *P*/2*a* = (*P* *P*_d_)^1/2^ < 100 W mm^−1^, then a compact first-order expression of *P*/*a* is derived,

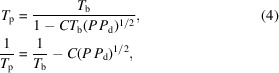

where *C* ≃ 6 × 10^−5^ to 8 × 10^−5^ mm W^−1^ K^−1^ is a constant parameter dependent on Si material properties at cryogenic temperatures. A very similar function is obtained for elliptical beam footprints. This simple dependence of *T*_p_ with the square root of absorbed power multiplied by power density has important consequences for the cooling of crystal monochromators. This will be further investigated in the remainder of the paper.

The slope error σ_SE_ caused by thermal deformation can be estimated using the linear thermal expansion Δ*L* of silicon at cryogenic temperatures and can be found elsewhere (Middelmann *et al.*, 2015 ▸). By assuming *T* ≃ *T*_p_ in the footprint area and *T* ≃ *T*_b_ at the depth *d* inside the crystal, we obtain

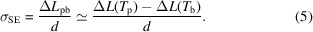

**Figure 1:**
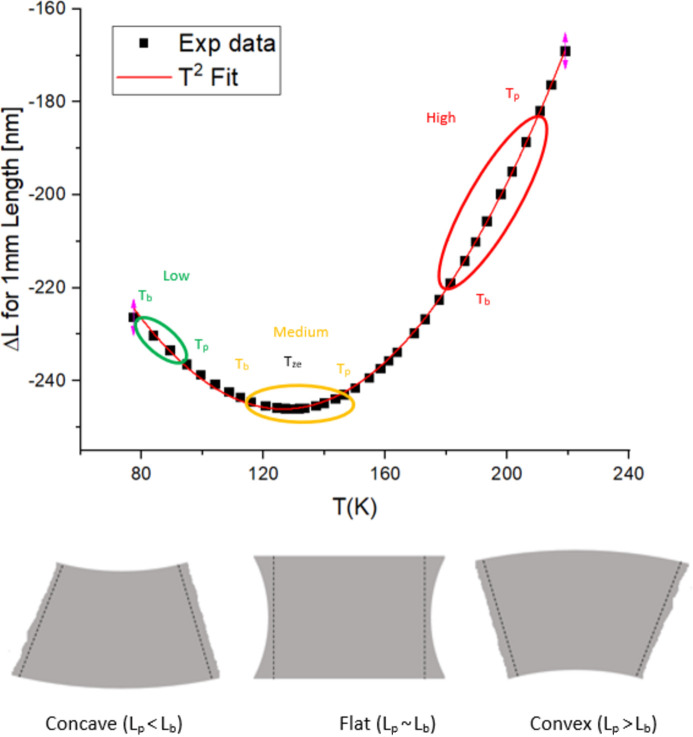
(Top) Thermal length expansion of a 1 mm Si crystal relative to room temperature (squares) fitted by a *T* ^2^ polynomial (red line). The coloured ellipses show examples of *T*_p_ and *T*_b_ corresponding to low-, medium- and high-power regimes (green, yellow and red, respectively). *T*_ze_ shows the temperature of minimum thermal expansion. (Bottom) Schematic representation of crystal deformation, switching from concave to flat, and to convex, when power is increased.

Δ*L*(*T*) can be approximated as a parabolic function of temperature as shown in Fig. 1 ▸,

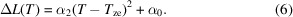

α_2_ and α_0_ are constants, and *T*_ze_ ≃ 127 K is the temperature of minimum thermal expansion of Si. A parabolic fit is sufficient compared with the previous fourth-order fitting (Khosroabadi *et al.*, 2022 ▸). Units for Δ*L* were changed to the more practical nanometres.

Therefore equation (5) can be re-written as

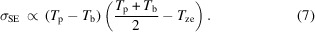

We define the second term in equation (7) as the *f* function,

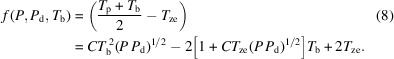


*f* is a universal function of cryo-cooled Si crystals, and the near-zero deformation conditions are found by setting it to zero. *f* has a complex dependence on *P* and *P*_d_, due to *T*_b_ [equation (3*c*)], and the solution will be given in Section 4.

Briefly, equation (8) can be solved for three different practical situations: (i) *P* and *P*_d_ change due to the ring current ramp, ID gap or insertion of filters; (ii) *P* changes due to changed white-beam slit aperture, while *P*_d_ is constant; (iii) *P* is constant while *P*_d_ changes due to Bragg angle variation for instance. Numerical solutions will be discussed in Section 4; however, in the latter case, the threshold power density *P*_d,c_, below which high deformation is prevented, is

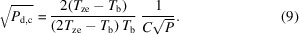




## 3. Power scenarios

The on-axis angular power emitted by an insertion device (ID) source is given by (Thompson, 2009 ▸)

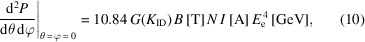

where *I* is the ring current. *B*, *N* and *K*_ID_ are the ID parameters, *i.e.* the magnetic field, the number of periods and the deflection parameter, respectively, and *G*(*K*_ID_) is a universal function with the value of >0.95 for *K*_ID_ > 1. The beam apertures typically used on hard X-ray beamline at DLS are Ω_H_ ≃ 140 µrad (horizontal) and Ω_V_ ≃ 60 µrad (vertical). On D-II these will reduce to Ω_H_ ≃ 80 µrad and Ω_V_ ≃ 60 µrad. These are about five to six times the photon beam r.m.s. divergence from source. The angular power density of an undulator source is nearly constant in these typical apertures, and we can derive

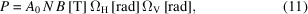




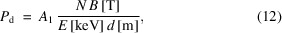

where *d* is the source-to-DCM distance and *E* is the monochromatic photon energy. The constants *A*_0_ and *A*_1_ and the power range are given in Table 1 ▸ for Si111 on the DLS and D-II machines. These are calculated at 4 mm (minimum) gap for 2 m-long CPMUs (*N* = 113, *K*_ID_ = 2.2, *B* ≃ 1.4 T) and HPMUs (*N* = 106, *K*_ID_ = 2, *B* ≃ 1.17 T). The power density is calculated assuming *d* = 30 m and energy from 2.1 to 25 keV. The data agree well with accurate calculation using *SPECTRA* (Tanaka, 2021 ▸; Tanaka & Kitamura, 2001 ▸); however, it should be noted that the power absorbed by the first crystal is about 10–14% lower than calculated, due to scattering processes (Zhang *et al.*, 2013 ▸). On several beamlines attenuation is performed by window and filter materials. The figures presented here are for the most severe power load scenarios. Finally, as in several other synchrotrons with upgraded photon sources, the issue is increased power density rather than total power. For instance, power density at lowest DCM energies of ∼2 keV will surpass ∼70 W mm^−2^ for a CPMU: this is the worst-case power scenario on D-II hard X-ray beamlines.

For beamlines exploiting high-magnetic-field insertion device (wiggler) photon sources, and accepting large horizontal fans, the total power will instead increase considerably. The beam footprint area will also be the same order of magnitude as the crystal size. The analytical treatment presented would not apply to such scenarios.

## 4. Model validation and threshold power

**Figure 2:**
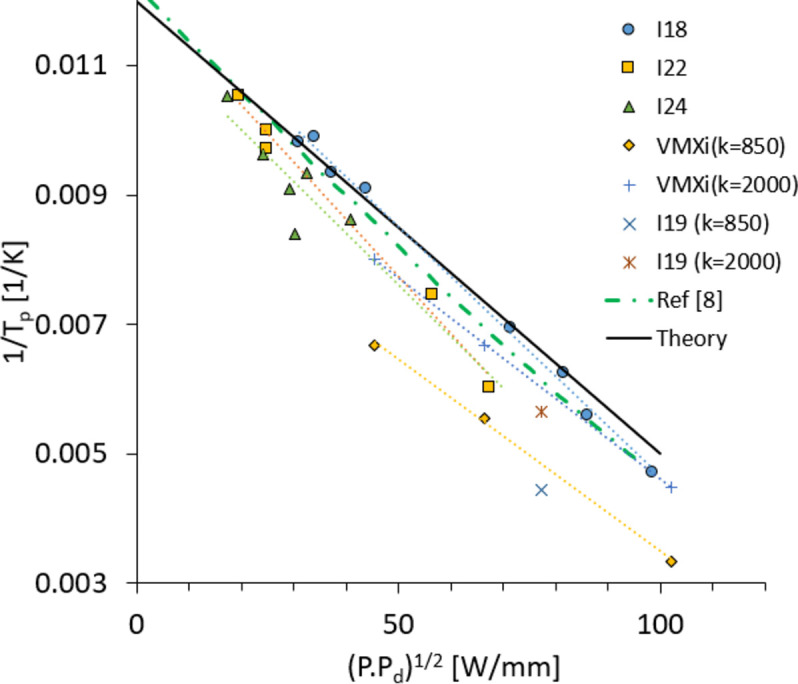
The black solid line is the inverse of peak temperature calculated with equation (4) using *C* = 7 × 10^−5^ mm W^−1^ K^−1^. The symbols are FEA results (see Table 2 ▸) and the green dashed line is from the literature (Zhang *et al.*, 2013 ▸). Other lines are guides to the eye.

Realistic power values on some upgraded DCMs are shown in Table 2 ▸. The inverse of the peak temperature calculated with equation (4) is plotted in Fig. 2 ▸ alongside FEA data from a variety of DLS and D-II scenarios. The symbols are FEA results for new DCMs installed in recent years on beamlines I18, I19, I22, I24 and VMXi with the parameters in Table 2 ▸. Previously published FEA data (Zhang *et al.*, 2013 ▸) are also plotted for comparison. The data show a linear trend as predicted by the model and in good agreement with FEA data. The simple relationship 1/*T*_p_ ∝ (*P* *P*_d_)^1/2^ well describes the physical problem of cryo-cooled Si crystals. The vertical offset is caused by different *T*_b_ values for different scenarios mentioned in Table 2 ▸. The slight deviation in slope is due to the dependence of the *C* parameter [equation (4)] on *P* and *P*_d_.

Schematic examples of deformation are given in Fig. 1 ▸. Generalized ranges of *T*_b_ and *T*_p_ at low, medium and high power are shown in this plot. The lengths of the coloured elliptical areas increase with power and power density as predicted by equation (4). The deformation, or slope error, is proportional to the effective temperature gradient in this diagram as per equation (6). The equation shows that both *T*_b_ and *T*_p_ contribute to the resulting thermal deformation. Crystal surface deformation is concave at low power (*T*_b_, *T*_p_ < *T*_ze_), nearly flat at medium power (*T*_b_ < *T*_ze_ < *T*_p_) and convex at high power (*T*_b_, *T*_p_ > *T*_ze_). Smallest deformation, σ_SE_ ≃ 0, is achieved for *T*_b_ and *T*_p_ temperature values that are symmetric relative to *T*_ze_. This optimum, medium power regime is the so-called ‘sweet spot’. The equation also illustrates that attempts to keep *T*_p_ close to *T*_ze_ do not work in practice. Normally this is achieved for higher *T*_p_, *e.g.* 150 K for *T*_b_ ≃ 95 K.

Slope error estimation *via* the present model matches FEA data (Khosroabadi *et al.*, 2022 ▸). However, more accurate data can be obtained by detailed FEA analysis in practice. Equations (6) and (8) describe the conditions for minimum σ_SE_, *i.e.* either at very low power or close to the threshold power. Excessive thermal expansion is indicated in Fig. 1 ▸ by the elongated red ellipse and is responsible for a rapid deformation regime. We define this as the threshold regime to be avoided.

**Figure 3:**
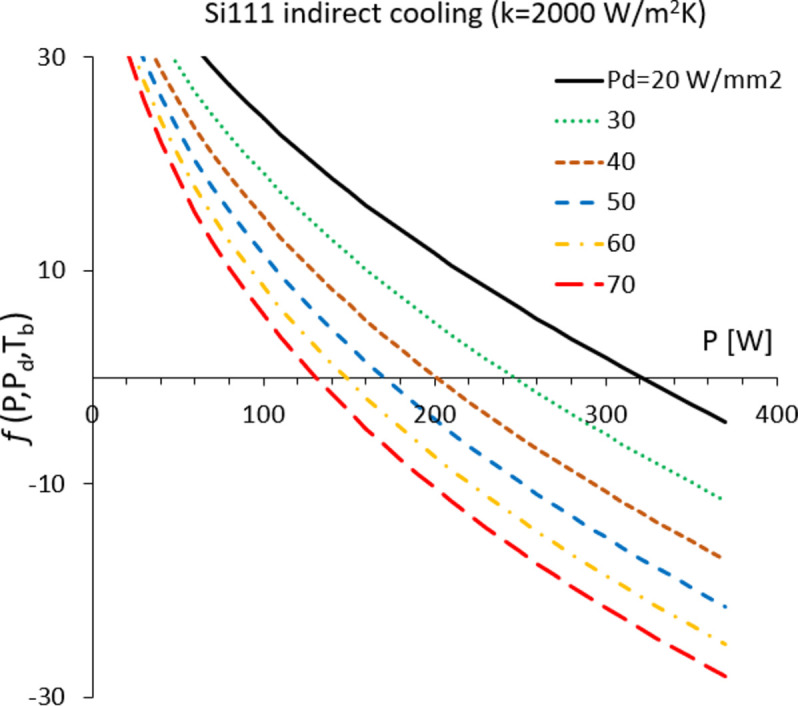
Function *f* for new DCM cooling geometry at DLS at several power density values.

**Figure 4:**
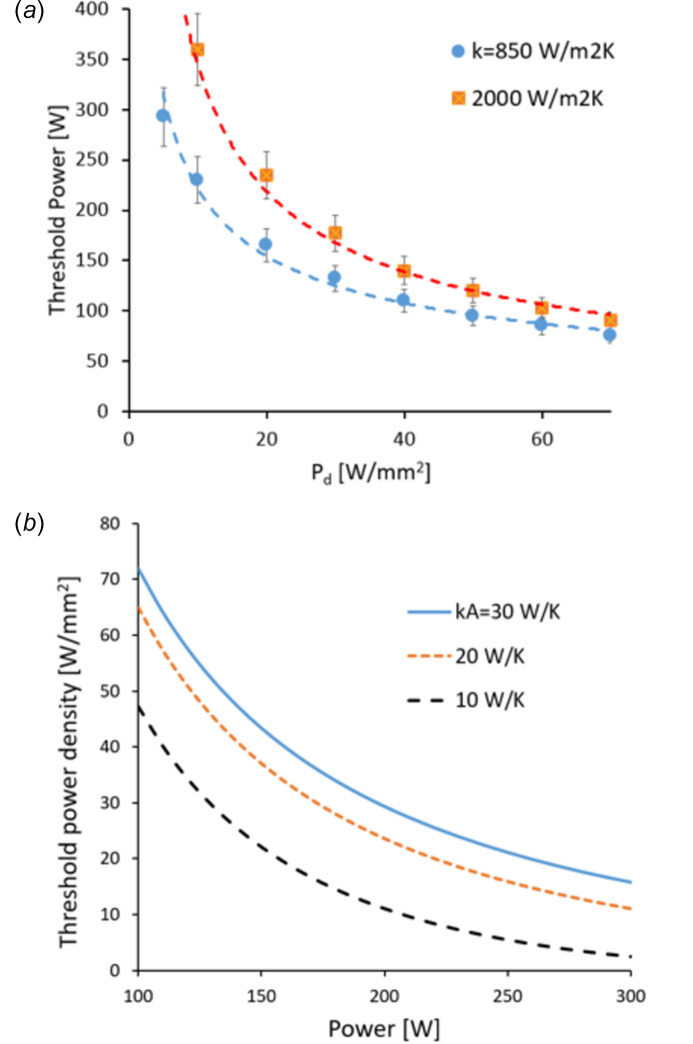
(*a*) Threshold power derived from Fig. 3 ▸ (symbols) and guidelines (dashed), as a function of the power density for two different values of thermal contact conductance. The ±10% error bar is also shown. (*b*) Threshold power density calculated from equation (9) for different values of *kA*.

**Figure 5:**
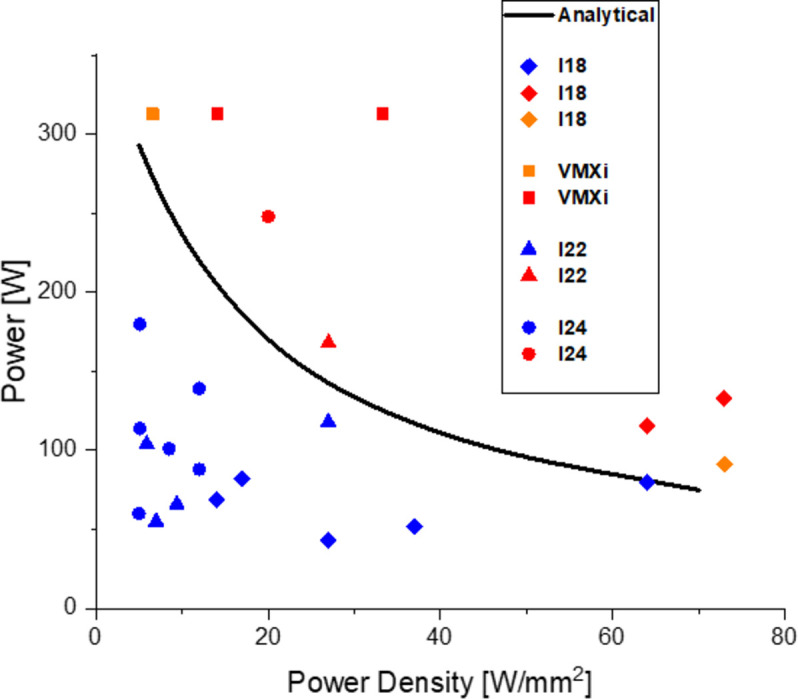
Threshold power scenarios defined in this paper (solid line). Symbols indicate FEA data from Table 2 ▸ power scenarios and beamline specified in the legend. Blue symbols indicate power levels leading to acceptable crystal slope errors; orange and red are for deformation levels leading to decreased optical performance, such as decreased diffraction efficiency or the lensing effect.

Recent DCMs at DLS (Sanchez-Navarro, 2021 ▸) have indirect side cooling and total contact area of *A* = 0.014 m^2^. Examples of the *f* function [equation (8)] for these are shown in Fig. 3 ▸. Threshold power is found at *f* = 0, and the results are plotted in Fig. 4 ▸(*a*), using some typical values for the contact conductance corresponding to low and average thermal cooling (*k* = 850 and 2000 W m^−2^ K, respectively). Threshold power density [equation (9)] is plotted in Fig. 4 ▸(*b*). These data and concepts are confirmed by FEA data performed for scenarios in Table 2 ▸. An acceptable degree of crystal deformation *S* was defined that would ensure conservation of photon beam brightness, spectral properties and focusing performance of the downstream optics. The result of this analysis is summarized in Fig. 5 ▸. The blue symbols represent power scenarios for which σ_SE_ calculated with FEA is <*S*. The red and orange symbols are for deformation at or above such a limit. The threshold power has an error bar due to the approximations used. Therefore, near these conditions the assessment should be more accurate.

**Figure 6:**
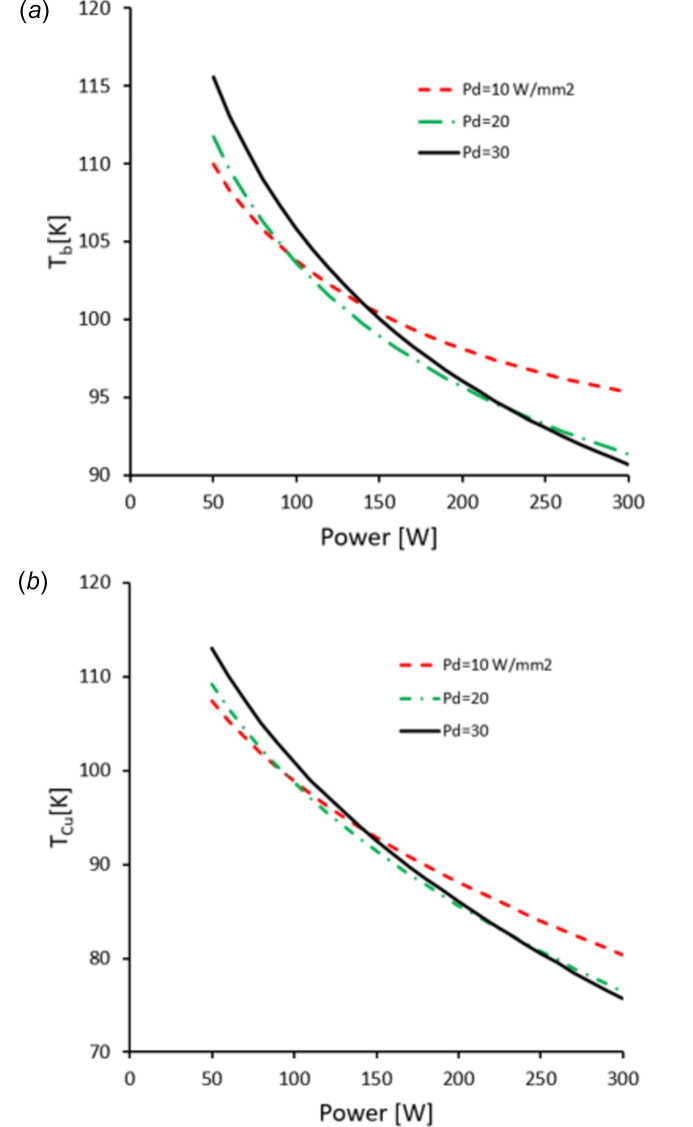
Optimum temperature for the Si crystal base (*a*) and the Cu block (*b*) as a function of power and power density, ensuring the lowest deformation.

Below the threshold, equation (8) predicts the optimum *T*_b_ temperature which minimizes the crystal surface deformation. This is shown in Fig. 6 ▸(*a*) and suggests that intentionally heating the crystal, or adjusting the flow rate of liquid nitro­gen, can bring the temperature close to the ‘sweet spot’ (Khosroabadi *et al.*, 2022 ▸; Sanchez-Navarro, 2022 ▸). The values plotted are for guidance only, as they depend on specific designs of cryo-cooled crystals. The copper block ‘sweet spot’ temperature can also be calculated from equation (3*c*), as shown in Fig. 6 ▸(*b*), using *kA* = 28 W K^−1^. These are very realistic values, being all above the boiling temperature of liquid nitro­gen at 77 K.

In summary, the threshold power curve is a tool that can be used to decide whether a set of *P* and *P*_d_ values are acceptable. The criterion has been used at DLS to choose suitable photon angular fan acceptance values, and to recommend additional filtering. The power regime above the threshold also represents a condition in which the deformation cannot be controlled as it increases steeply with increased power.

## 5. Summary and conclusion

A theoretical model has been developed to calculate the temperature distribution and surface deformation of an indirectly cryo-cooled Si crystal. Setting the conditions for lowest deformation leads to the definition of a threshold power level, above which the crystal deformation is un­acceptably high. One practical result is the possibility to control the diffracted X-ray beam divergence or focal spot size at the sample position via intelligent cooling, keeping the crystal temperature within a small and well defined range. It has been shown that two characteristic temperatures, the peak and the base temperatures, have a unique relationship with (*P* *P*_d_)^1/2^. FEA data have confirmed this behaviour. The threshold power curve is a function of contact conductance, crystal base temperature, power and power density. The model described here can be adapted to different optics geometries.

We propose to use this model as an intuitive and fast method to understand and limit (by improved designs) thermal deformation in cryo-cooled Si crystals.

## Figures and Tables

**Table 1:** Values for the *A*_0_ and *A*_1_ coefficients on the DLS machine (*E*_e_ = 3 GeV) and D-II (*E*_e_ = 3.5 GeV) for *I* = 300 mA; maximum power *P* (W) and power density range *P*_d_ (W mm^−2^) for CPMU and HPMU sources are given

	*A*_0_	*A*_1_	CPMU		HPMU	
			*P*	*P*_d_	*P*	*P*_d_
DLS	250	494	330	3–41	260	2–32
D-II	460	909	350	7–76	275	5–60

**Table 2:** List of FEA calculations for different source types and several beamlines (the symbols are the same as in the legend of Fig. 5 ▸)

Beamline	Machine	Undulator source	DCM acceptance (µrad)	*E* (keV)	Power (W)	Power density (W mm^−2^)	Symbols for legend in Fig. 5 ▸
I18	DLS	U27	64 × 43	2.34	43	27	Blue diamond
		HPMU19.5	64 × 43	2.05	52	37	Blue diamond
	D-II	HPMU19.5	60 × 60	2.05	133	73	Red diamond
			50 × 50	2.05	91	73	Orange diamond
			50 × 50	8.0	82	17	Blue diamond
		CPMU21	60 × 60	2.05	116	64	Red diamond
			50 × 50	2.05	80	64	Blue diamond
			50 × 50	8.0	69	14	Blue diamond
I22	DLS	U25	80 × 50	6.71	55	7	Blue triangle
		HPMU18.7	80 × 50	6.0	66	9.4	Blue triangle
	D-II	HPMU18.7	60 × 60	6.0	168	27	Red triangle
			50 × 50	6.0	118	27	Blue triangle
			50 × 50	25.0	104	5.9	Blue triangle
I24	DLS	U21	80 × 43	6.0	60	5	Blue circle
		CPMU17.6	80 × 43	6.0	101	8.5	Blue circle
	D-II	CPMU17.6	60 × 60	6.0	248	20	Red circle
			50 × 50	8.0	139	12	Blue circle
			40 × 40	8.0	88	12	Blue circle
			50 × 50	25.0	180	5.1	Blue circle
			40 × 40	25.0	114	5.1	Blue circle
VMXi	D-II	CPMU17.6	75 × 58	5.6	313	33.3	Red square
			75 × 58	13.2	313	14.1	Red square
			75 × 58	28.2	313	6.6	Orange square
